# Michigan State Board of Health

**Published:** 1886-01

**Authors:** 


THE MICHIGAN STATE BOARD OF HEALTH,

In addition to its previous circulars on the Prevention of the Introduction of Communicable Diseases—has issued a special order (Circular 93) containing the following rules to be enforced in the transportation of dead bodies :

RULE 1. Before being placed in the coffin, the remains of the deceased shall be wrapped in a sheet thoroughly saturated with a strong solution of chlorinated soda, or chloride of zinc, one-half pound to the gallon of water, or a solution made in proportions as follows : water, one gallon; sulphate of zinc, eight ounces; common salt, four ounces.

RULE 2. The coffin shall be packed in a strong box, and shall be surrounded by sawdust, saturated with zinc solution of a strength equal to that required by Rule 1, above

RULE 3. The body shall not be accompanied by persons who (or articles which) have been exposed to the infection of the disease.

RULE 4. The coffin shall not be opened, neither shall the box be opened at the place of destination.

RULE 5. The burial shall take place immediately after arrival at the place of destination.

